# Pre-Activation Negativity (PrAN) in Brain Potentials to Unfolding Words

**DOI:** 10.3389/fnhum.2016.00512

**Published:** 2016-10-10

**Authors:** Pelle Söderström, Merle Horne, Johan Frid, Mikael Roll

**Affiliations:** ^1^Centre for Languages and Literature, Lund UniversityLund, Sweden; ^2^Humanities Laboratory, Lund UniversityLund, Sweden

**Keywords:** ERP, pre-activation, prediction, speech processing, lexical competition, word-initial fragment, PRAN

## Abstract

We describe an event-related potential (ERP) effect termed the “pre-activation negativity” (PrAN), which is proposed to index the degree of pre-activation of upcoming word-internal morphemes in speech processing. Using lexical competition measures based on word-initial speech fragments (WIFs), as well as statistical analyses of ERP data from three experiments, it is shown that the PrAN is sensitive to lexical competition and that it reflects the degree of predictive certainty: the negativity is larger when there are fewer upcoming lexical competitors.

## Introduction

In spoken language processing, listeners have to be able to quickly access the meaning of words in a stream of speech which unfolds rapidly over time. To achieve this, it has been shown that listeners take advantage of cues that can be used to predict upcoming information, such as specific words or prosodic structures (DeLong et al., 2005; van Berkum et al., 2005; Breen et al., 2014; Norris et al., 2016). Even before hearing a complete word, listeners are thought to fully or partially pre-activate competing similar-sounding candidates that constitute candidates for whole words (e.g., Morton, 1969; Luce, 1986; McClelland and Elman, 1986; Marslen-Wilson, 1987; Norris, 1994; Pierrehumbert, 2003). In Swedish, listeners use word melodies called “word accents” to predict—already at the beginning of a word—how it will end (Roll et al., 2010, 2013, 2015; Söderström et al., 2012, 2016; Roll, 2015). In event-related potential (ERP) studies, word stems with one of the word melodies (accent 1) have produced increased negativity as compared to stems with the other melody (accent 2). Since accent 1 is associated with fewer possible continuations than accent 2, we hypothesize that the negativity found for word beginnings is related to certainty about the end of the word. The negativity produced by accent 1 stems seems to be associated with increased neural activity and correlates with a decrease in response times to tasks focusing on word-final suffixes (Roll et al., 2015). We therefore propose that it indexes pre-activation of the word ending, i.e., that it is a “pre-activation negativity” (PrAN). To test this hypothesis, the present study investigates whether there is a gradual relation between PrAN amplitude and possible certainty about word endings—as defined by a lexical competition measure—based on information in the phonological structure of word beginnings.

Most studies on anticipation mechanisms in language processing have focused on behavioral and neurophysiological indices of violated predictions, prediction error or surprisal (e.g., DeLong et al., 2005), most often for words expected in a certain context. The present study, however, investigates an effect which is hypothesized to index the *pre-activation* of anticipated material before it is encountered, i.e., that it reflects pre-activation *as it happens*. Furthermore, we hypothesize that this pre-activation mechanism can operate within words, starting from the word-initial fragment (WIF). WIF—as defined in the present study—consists of the first speech sounds of a word including the first vowel, a unit containing important segmental and suprasegmental (prosodic) information about a word’s structure.

It can be assumed that an important factor determining certainty about a word’s ending is how many possible continuations a WIF has, something which is reflected in its number of lexical competitors. In order to test the hypothesis that the PrAN is sensitive to the number of lexical competitors, we used a lexicon database of Swedish to investigate the relationship between the number of competing words that begin with a particular WIF and the amplitude of the ERP elicited by that fragment. The assumption is that WIFs with few continuations, i.e., those which can be followed by a smaller number of grammatical or lexical morphemes, lead to less lexical competition. Hence, these WIFs associated with fewer lexical competitors, including fewer endings, should have greater pre-activation potential and consequently elicit larger PrAN amplitudes.

### WIFs and Swedish Word Accents

The WIFs analyzed here constitute the beginnings of word stems. In Central Swedish, each word stem carries a tone which is either low (“accent 1”) or high (“accent 2”). The tone is realized on the stressed vowel (Bruce, 1977, 1986). The same stem can be pronounced with one of the two different tones depending on what follows. For example, the singular noun suffix *-en* is always preceded by an accent 1 stem tone (e.g., *båt*_1_*-en* “the boat”, where the subscript number indicates accent 1 or 2), while the plural noun suffix *-ar* is always preceded by accent 2 (e.g., *båt*_2_*-ar* “boats”). In the present study, the initial segments up to and including the vowel bearing the tone, i.e., *bå*_1_- and *bå*_2_-, will be analyzed as WIFs.

All productive compounds have accent 2 on their initial constituents, such as *båt*_2_*-hus* (“boat house”). Since compounding can—in principle—create an unlimited number of words, this would imply that lexical competition is greater in WIFs with accent 2. Thus, if a WIF associated with a smaller number of lexical competitors has the potential to more strongly pre-activate a limited number of possible continuations, then WIFs with accent 1 should on average have larger pre-activation potential. In fact, as we show in this study, accent 2 WIFs are on average associated with 11 times as many word-internal continuations as accent 1 WIFs, something which should have a considerable effect on the pre-activation of possible upcoming candidates.

### Pre-Activation Negativity (PrAN)

In ERP studies, accent 1 has been found to give rise to a slightly left-lateralized increased negativity over frontocentral electrode sites at 136–280 ms as compared to accent 2^1^ (Roll et al., 2010, 2013, 2015; Roll, 2015; Söderström et al., 2016). It has been shown that the effect cannot be explained by the acoustic difference between the tones or by the lexical status of the stem, i.e., whether it is a pseudo-stem or an existing stem (Roll et al., 2013; Roll, 2015; Söderström et al., 2016). Instead, we have hypothesized that the observed ERP effect reflects activation of the upcoming suffix rather than simply perception of the tone. Various lines of evidence suggest that this could indeed be the case. To begin with, the amplitude of the negativity has been found to correlate with the degree to which response times to suffixed words are facilitated by accent 1 as a cue to an upcoming suffix (Roll et al., 2015). An explanation for this correlation could be that accent 1 more strongly pre-activates upcoming word-internal material, since it is only associated with a limited set of suffixes, whereas accent 2 appears in all compound words in addition to a set of suffixed words. As noted above, due to their presence in productive compound words, accent 2 WIFs are associated with almost 11 times as many continuations as compared to accent 1 WIFs.

Accent 1 stems have also been observed to elicit more neural activity as measured by global root mean squares (gRMS) and blood-oxygen-level dependent signal (BOLD; Roll et al., 2015). Roll et al. (2015) analyzed ERP and fMRI data from identical test paradigms and found that fMRI activation in an area which has been associated with morphological processing (left inferior frontal gyrus, LIFG; Brodmann area 47) correlated with ERP effects in the 136–280 ms window, occurring even before the suffix had been heard. Similarly, González-Garcia et al. (2015) also found that expectations about an upcoming stimulus can initiate a linguistic pre-activation process in the LIFG. This might indicate that the participants in Roll et al. (2015) had accessed morphological information before they perceived the suffix, pointing to some type of pre-activation mechanism indexed by the ERP negativity which is the focus of investigation in the present study.

Further support for the pre-activation hypothesis comes from Söderström et al. (2016), a study in which participants were asked to judge whether a word was singular or plural based on suffixes attached to pseudoword stems with either accent 1 or 2. In a critical condition, suffixes were replaced with a light cough. Correlation analyses showed that participants with larger pre-activation negativities for accent 1 had higher response accuracy in judging the meaning of the masked suffix. Furthermore, cough-endings gave rise to a P3a component which correlated positively with PrAN amplitude, suggesting that listeners were more surprised by a masked suffix when that suffix had been strongly anticipated based on the word accent. Taken together, previous results suggest that PrAN correlates with a mechanism with which listeners can commit to an expected suffix before that ending has been heard.

A possible neuropsychological interpretation of the process underlying the hypothesized PrAN is that it reflects activation of cortical memory traces of an upcoming lexical item, such as a suffix (Roll et al., 2015; Söderström et al., 2016; see Pulvermüller and Shtyrov, 2003). In choosing between fewer candidate items—such as those occurring after accent 1—the pre-activation mechanism can commit more strongly to a likely candidate and thus more strongly activate its memory traces before the item is actually perceived. It has previously been suggested that a template or memory trace can be activated prior to presentation of a strongly predicted stimulus (SanMiguel et al., 2013; Foucart et al., 2015).

In summary, the negativity which has earlier been found for accent 1 might be a general index of how strongly upcoming word-internal morphemes are pre-activated. This forms the basis of the hypothesis tested in the present study. A test implication of this hypothesis is that there should be a gradual relationship between the amplitude of the negativity associated with a WIF and the predictive certainty as regards how a word will end. Thus, decreased lexical competition should facilitate pre-activation and lead to an increase in the proposed PrAN effect.

### Lexical Competition and PrAN

Most models of speech recognition assume that similar-sounding words enter into competition with each other at some point during the recognition process (e.g., Luce, 1986; McClelland and Elman, 1986; Marslen-Wilson, 1987; Norris, 1994; Luce et al., 2000). It has been found that listeners are slower to process words with many similar-sounding competitors, often referred to as phonological “neighbors” (Luce and Pisoni, 1998; Vitevitch and Luce, 1998, 1999). As an example of this, it has been found that words which begin with the same initial segments—such as *candle* and *candy—*compete with each other before the words can be clearly disambiguated (e.g., Allopenna et al., 1998). WIFs which cue a large set of competitors could therefore be assumed to be processed differently from those with fewer competitors. For instance, a number of ERP studies have found that words with fewer similar-sounding competitors elicit more negative-going ERPs within 250 ms of spoken word onset, as compared to words with more competitors (e.g., Dufour et al., 2013; Hunter, 2013). It has also been found that grammatical constraints can influence lexical competition and access (Strand et al., 2014), making it possible to argue that the tone-suffix connection in Swedish words constitutes a morphophonological “micro-context” which can be used to constrain possible word-internal continuations. In other words, when possibilities for continuation are restricted—as in the case of WIFs with accent 1—the speech processing mechanism can be more accurate in its predictions and thus begin to selectively pre-activate highly likely candidates (see DeLong et al., 2005; Gagnepain et al., 2012; Dikker and Pylkkänen, 2013).

### The Present Study

The present study tested the hypothesis that WIFs give rise to a PrAN reflecting the degree of pre-activation of expected continuations of the word. We investigated the correlation between ERP data where an assumed PrAN has been observed and the number of words that enter into competition following a given WIF in the test words. Using WIF-based lexical competition measures and statistical analyses (within-subjects analysis of variance (ANOVA) and linear regression analysis), we analyzed ERP data from three previous studies (Roll et al., 2015 (henceforth referred to as “Experiment 1”); Roll et al., 2010 (“Experiment 2”); Roll, 2015 (“Experiment 3”)) in which participants listened to sentences with embedded target words composed of different word stems associated with accent 1 or accent 2 and an inflectional suffix, either singular or plural. All sentences had been designed so that there were no contextual expectations for accent 1 or 2 WIFs.

The hypothesis was that increased potential for pre-activation—i.e., decreased lexical competition—would lead to greater PrAN amplitudes in the 136–280 ms time window which, as noted above, has previously been found to correlate with fMRI effects related to morphological processing of upcoming suffixes (Roll et al., 2015). WIFs which cue rather many words, such as compounds, can be expected to have connections to relatively large numbers of competing lexical forms. WIFs with fewer lexical competitors, however, can be expected to have stronger connections with their relatively limited number of connected forms. On the basis of previous research results (see “Pre-Activation Negativity (PrAN)” Section), this lower degree of lexical connectivity would therefore be expected to be reflected in an increased ERP negativity effect. This effect was, furthermore, expected to be gradual, so that the amplitude of a WIF’s ERP negativity could be expressed as a continuous linear function of lexical competition.

We defined the degree of “lexical competition” in the present study as *the number of words which share a given WIF*, where WIF is defined as the initial segments and prosodic features of a word, up to and including the (stressed) vowel (see “onset similarity” in Dufour et al. (2013) or “onset density” in Vitevitch (2002)). A majority of the WIFs analyzed in the present study—e.g., *bå*- from *båt—*had a simple consonant-vowel (CV or CCV) onset structure, which corresponds roughly to the 2–3 initial phonemes thought to activate candidates during lexical access (Marslen-Wilson and Welsh, 1978; Marslen-Wilson and Tyler, 1980; Norris, 1994). Lexical competition data underwent log-transformation prior to analysis.

## Materials and Methods

ERP and lexical competition data from three studies were used for the analyses.

### Stimuli

The auditory stimuli used to elicit the ERP responses in the present study were disyllabic words (word stem followed by a singular or plural suffix) embedded in sentences. There were no cues as to which suffix was coming up before the WIF. Rather, it was not until the WIF had begun to be perceived that test participants could begin anticipating how the word could unfold. An example sentence from Roll et al. (2015) is *Rut fick fisken/fiskar till lunch* (“Rut got fish-singular/fish-plural for lunch”). The WIF in this particular example is *fi-*. In Experiments 1 and 3, the task was to judge whether the word was singular or plural, while Experiment 2 used a sentence-level semantic acceptability judgment task. The disambiguation point for judging which suffix the participant had heard was the onset of the singular or plural suffix. Mean duration from WIF onset to suffix onset in all three experiments was 472 ms (*SD* = 56). In Experiment 1, accent 1 WIFs had a mean duration of 428 ms (*SD* = 60) and accent 2 WIFs a duration of 424 ms (*SD* = 67). The corresponding data for Experiment 2 was 656 ms (*SD* = 49) for accent 1 WIFs and 642 ms for accent 2 WIFs (*SD* = 62). In Experiment 3, accent 1 WIFs had a mean duration of 335 ms (*SD* = 54) and accent 2 WIFs had a mean duration of 345 ms (*SD* = 45). There were no lexical cues to the word-internal continuation prior to WIF onset, and the initial portions of the sentences were identical across conditions. Thus, the point where listeners could begin to pre-activate the suffix was the very beginning of the WIF. Average suffix duration in Experiment 1 was identical for accent 1 and 2 suffixes, 241 ms (*SD* = 24 ms). In Experiment 2, accent 1 suffixes (*M* = 251 ms, *SD* = 25) were slightly longer than accent 2 suffixes (*M* = 217 ms, *SD* = 30). Accent 1 suffixes in Experiment 3 (*M* = 215 ms, *SD* = 28) were shorter than accent 2 suffixes (*M* = 243 ms, *SD* = 53).

### ERP Data

High-pass filters of 0.05 Hz and low-pass filters of 30 Hz were applied in all three studies. Pre-stimulus baselines of 200 ms were used. Independent component analysis (ICA) was used for ocular artifact correction, and epochs where the electroencephalography (EEG) amplitude exceeded ±100 μV were rejected. For Experiments 1 and 3, which used the same electrode setup, the regions-of-interest (ROIs) used were left anterior (F7, F3), right anterior (F4, F8), left central (T7, C3), right central (C4, T8), left posterior (P7, P3) and right posterior (P4, P8). For Experiment 2, ROIs were left anterior (F7, F5, F3, FT7, FC5, FC3), mid anterior (F1, FZ, F2, FC1, FCZ, FC2) right anterior (F4, F6, F8, FC4, FC6, FT8), left central (T7, C5, C3, TP7, CP5, CP3), mid central (C1, CZ, C2, CP1, CPZ, CP2), right central (C4, C6, T8, CP4, CP6, TP8), left posterior (P7, P5, P3, P07, PO5, O1), mid posterior (P1, PZ, P2, PO3, PO4, OZ) and right posterior (P4, P6, P8, PO6, PO8, O2).

For the ERP data, the average amplitude in the time window of 136–280 ms measured from fundamental frequency (F0) onset used in Roll et al. (2015)—“Experiment 1”—was assumed for the PrAN. To ascertain this, we investigated the PrAN effect in the same time window in the Roll et al. (2010) data (“Experiment 2”) and the Roll (2015) data (“Experiment 3”). In the Roll (2015) data, the negativity was found to occur slightly later, leading us to focus the analysis on the later part of the window (220–280 ms). Experiments 1 and 3 used the same stimulus words, but these datasets were analyzed separately, as Experiment 3 investigated South Swedish rather than Central Swedish^2^. ERPs^3^ for each WIF in the studies were extracted with pitch onset as zero point. Since a small number of words began with the same WIF, e.g., *ko-ck* (“chef”) and *ko-pp* (“cup”), ERP responses were averaged across these identical fragments.

### Calculating Lexical Competition—Lexicon Database

In order to obtain an estimate of the lexical competition for a given WIF, we used a freely available lexicon database for Swedish, the NST lexicon (see Andersen, 2011). The lexicon database is a large full-form lexicon for Swedish linking text words to their canonical pronunciations. The lexicon consists of one single text file. Each line in the file corresponds to an entry in the lexicon and consists of 51 fields separated by semicolons. The fields contain information about the entry, such as pronunciation. There are 927,167 items in the lexicon, of which some are automatically generated inflections. About 25% of the pronunciation entries have been manually checked. The lexicon contains information about phonetic segments, stress, word accents, syllable boundaries and sub-word boundaries of compound words, as well as syntactic information such as word class. In total, the lexicon uses 41 phonetic symbols for the pronunciation information. The lexicon allowed us to calculate the number of words that begin with a phonetically specified WIF and which also comply with other desired properties, such as word class and word accent. The result is the raw score of the lexical competition of a WIF.

**Figure 1:**
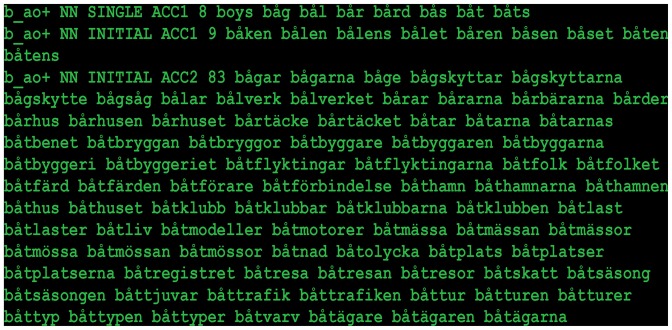
**Example of lexical competitors for the word-initial fragment (WIF) *bå* [bo:] (coded as *b_ao+* in the lexicon).** On each line, going from left to right, the fields (separated by space) contain WIF, word class (NN = noun), syllable position, word accent and number of competitors. This is followed by a list of the competitors. It was found that accent 2 WIFs are associated with almost 11 times as many continuations as accent 1 WIFs. A demo version of the database is available at http://person2.sol.lu.se/JohanFrid/webapps/syllable-lex/syllable-lex.php.

The vocabulary of any language is—in principle—infinite, but the number of words actually included in the lexical database must be finite. Thus, the actual choice of words may be somewhat arbitrary and the lexicon may contain words with very low usage, potentially introducing some undesired noise in the lexical competition scores. In order to somewhat mitigate this arbitrariness, we applied a frequency threshold to the lexicon. We matched the lexical database with a word frequency list based on the Swedish PAROLE^4^ corpus, and in the final version of the database, we only included words that had a PAROLE frequency of 2 or greater. In this way, we weigh in actual word usage. An example of a calculation of the number of words that begin with the WIF *bå-* associated with accent 1 (ACC1) or 2 (ACC2) is shown in Figure 1.

We chose to restrict our analysis to polysyllabic nouns with initial-syllable stress, since this was the category of words used in our recent studies on word accent processing, and since word accents are only associated with stressed syllables. There were 26 unique WIFs in Experiments 1 and 3 and 36 in Experiment 2. Since each WIF occurred with both accent 1 and 2, there was a total of 52 fragments in Experiments 1 and 3 and 72 in Experiment 2.

### Statistical Analyses of ERP Data and Lexical Competition Measurements

Two statistical analyses were carried out on the data: a within-subjects ANOVA and a linear regression analysis. The initial step was to conduct an ANOVA using the factor Competitors (few/many competitors) on the data from all experiments to test whether there was any significant difference in mean ERP amplitude for low and high lexical competition groups. Importantly, this allowed us to investigate which electrode sites should be used for the subsequent regression analyses. To achieve this, the WIFs were ranked according to the number of word forms which began with the particular fragment. The list was then divided into a low and a high lexical competition group using median split.

For the linear regression analyses, the data from Experiments 1, 2 and 3 were standardized to *z*-scores using mean ERP amplitudes and the standard deviation from each study separately (Experiment 1: ERP amplitude *M* = −2.44 μV, *SD* = 0.91 μV; Experiment 2: ERP amplitude *M* = −1.46 μV, *SD* = 1.36 μV; Experiment 3: ERP amplitude *M* = −2.15 μV, *SD* = 1.42 μV). This made it possible to present the data in one graph, and made the results from the three experiments more easily comparable. We then conducted regression analyses separately on each dataset in order to investigate whether any group difference found in the ANOVAs could also be expressed as a gradual relationship between lexical competition and ERP amplitude. The dependent variable was standardized PrAN amplitude (*z*-scores) and the independent variable was the *z*-score of lexical competition as defined above.

#### Testing Potential Effects of Lexical Frequency on PrAN

Any account of the proposed PrAN would have to explain why a similar effect has been found for accent 1 pseudo-stems followed by existing suffixes (Söderström et al., 2016). By definition, pseudo-stems do not have any lexical frequency but could be argued to enter into lexical competition based on the word accent and anticipated endings, such as suffixes (grammatical morphemes) or compounds (lexical morphemes). However, to exclude the possibility that any effects found were driven by lexical (token) frequency, we first performed regression analyses involving both lexical competition and token frequency. One-tailed *t*-tests were used to evaluate the significance of lexical competition. These analyses were followed by simple regression analyses on *z*-scores of lexical competition, allowing for easier comparison between the three experiments.

## Results

### Lexical Competition: Word-Initial Fragments (WIF)

WIFs were divided into a low and a high lexical competition group using median split. In Experiment 1, the lexical competition group averages for the ANOVA were 20.4 (*SD* = 11.4) words for the low competition group and 252.8 (*SD* = 195.0) for the high group. In Experiments 1 and 3, 85% of WIFs in the low competition group had accent 1. For Experiment 2, the averages were 13.6 (*SD* = 8.2) words for the low group and 227.8 (*SD* = 181.2) for the high group. In Experiment 2, 81% of WIFs in the low competition group had accent 1.

For accent 1, the total average number of continuations to WIFs in Experiments 1 and 3 was 24.8 (*SD* = 18.7) words and for accent 2, 248.5 (*SD* = 200.0). The corresponding data for Experiment 2 were 18.5 (*SD* = 15.6) for accent 1 and 223.0 (*SD* = 186.3) for accent 2. Thus, on average, WIFs with accent 2 were found to be associated with almost 11 times as many possible word-internal continuations as compared to accent 1 WIFs. The same was found to be true for the entire lexicon, where accent 2 WIFs were associated with 10.5 times as many continuations as accent 1 WIFs.

### Lexical Competition ANOVA: Experiment 1

**Figure 2:**
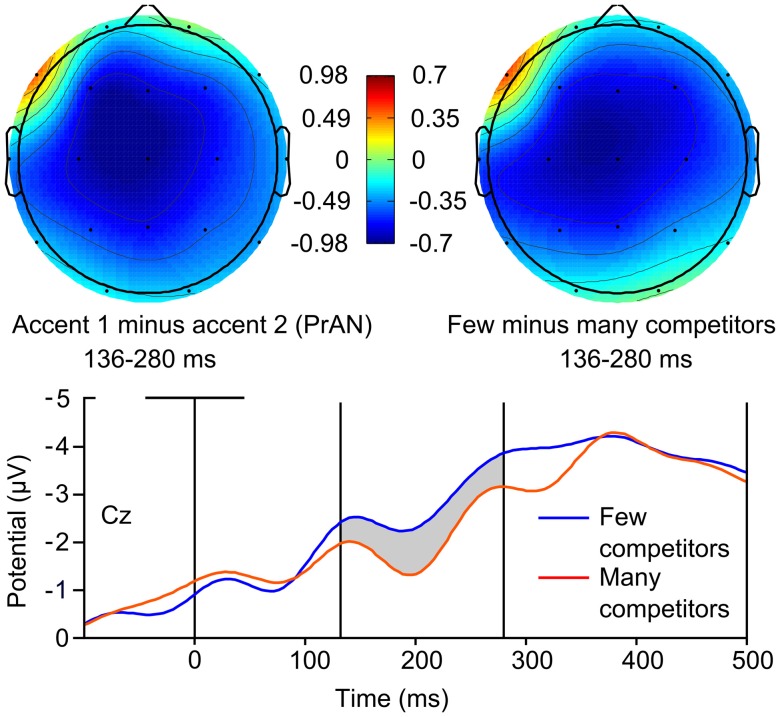
**Top left: event-related potential (ERP) scalp distribution of the pre-activation negativity (PrAN) effect found for accent 1 stems in Roll et al. (2015).** Top right: ERP scalp distribution calculated from the lexical competition analysis of variance (ANOVA) for the same data. Bottom: averaged ERPs from the Roll et al. (2015) data for WIFs with few (blue line) and many (red line) competitors.

In order to investigate possible effects of lexical competition on ERP amplitude, and in which electrode regions these effects could be seen, we conducted an ANOVA with the factor Competitors (few, many) as well as the same topographical factors as in Roll et al. (2015): Antpost (anterior, central, posterior) and Laterality (left, right). The dependent variable was average ERP amplitude in the 136–280 ms window using a pre-F0 onset baseline correction as in Roll et al. (2015). A Competitors × Antpost × Laterality interaction (*F*_(2,34)_ = 6.349, *p* = 0.009, $\eta_{p}^{2}$ = 0.272) was found, along with a marginal Competitors × Antpost interaction at left channels (*F*_(2,34)_ = 2.937, *p* = 0.088, $\eta_{p}^{2}$ = 0.147). ERP amplitude was significantly more negative for fragments with less lexical competition at left central (*F*_(1,17)_ = 6.868, *p* = 0.018, $\eta_{p}^{2}$ = 0.288) and left posterior (*F*_(1,17)_ = 4.850, *p* = 0.042, $\eta_{p}^{2}$ = 0.222) electrode sites. In Figure 2, the spatial scalp distribution of the negative effect seen in the ANOVA is compared to the topographic plot of the accent 1 negativity from Experiment 1. Figure 2 also shows the averaged ERPs for fragments with few and many competitors.

### Lexical Competition ANOVA: Experiment 2

**Figure 3:**
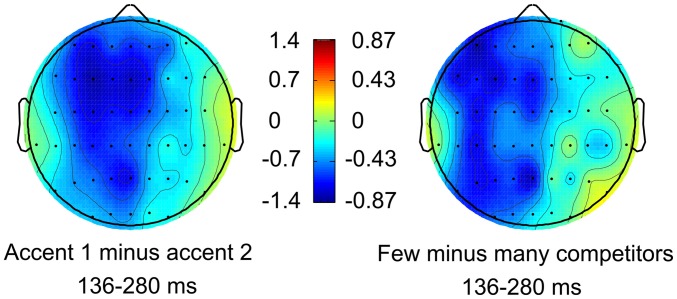
**Left: ERP scalp distribution of the PrAN effect found for accent 1 stems in Roll et al. (2010).** Right: scalp distribution of the negative ERP amplitude obtained from the lexical competition ANOVA for WIFs with few competitors in Experiment 2.

A lexical competition ANOVA was also performed on Experiment 2 data in the 136–280 ms time window in order to investigate which electrode regions could be used for the subsequent regression analysis. A Competitors × Laterality interaction was found (*F*_(2,38)_ = 3.831, *p* = 0.032, $\eta_{p}^{2}$ = 0.168) as well as a main effect of Competitors at left-lateralized electrode sites (*F*_(1,19)_ = 4.410, *p* = 0.049, $\eta_{p}^{2}$ = 0.188, see Figure 3). ERPs for WIFs with few competitors were thus significantly more negative at left-lateralized electrode sites.

### Lexical Competition ANOVA: Experiment 3

A lexical competition ANOVA was performed to see whether there was a difference between WIFs with many or few lexical competitors in the time window and electrode regions where a PrAN had been found in Experiment 3. A significant main effect of Competitors was found, showing that ERPs for WIFs with many competitors were more negative at 220–280 ms over left anterior and left central channels (*F*_(1,19)_ = 26.835, *p* < 0.001, $\eta_{p}^{2}$ = 0.585).

### Regression Experiment 1

To investigate the possibility that the effect of lexical competition on the PrAN could be expressed as a gradual function, we performed linear regression analyses on the ERPs at the electrode sites that had a significant effect in the ANOVAs. Thus, the left central and left posterior electrode sites were used to conduct a regression analysis on the data from Experiment 1. We included lexical frequency as a predictor in order to investigate its influence on PrAN amplitude. A significant regression equation was found in the 136–280 ms window (*F*_(2,101)_ = 8.852, *p* < 0.001, *R*^2^ = 0.149). Lexical frequency did not have a significant effect (*t* = 0.676, *p* = 0.501), but an effect was found for lexical competition (*t* = 2.437, *p* = 0.009). This led us to investigate lexical competition alone in a simple regression analysis. A significant equation was found (*F*_(1,50)_ = 8.832, *p* = 0.003, *R*^2^ = 0.150). The ERP negativity increased 0.387 standard deviations for each unit decrease of lexical competition. The function for the predicted ERP amplitude is shown in example 1.

(1) PrAN = 0.387 (lexical competition)

### Regression Experiment 2

Focusing on the significant electrode sites with the largest PrAN amplitude difference (Figure 3), we used left-lateralized channels for the 136–280 ms time window in the regression analysis of the data from Experiment 2. The regression involving both lexical frequency and competition was significant (*F*_(2,213)_ = 8.224, *p* < 0.001, *R*^2^ = 0.072), but again, only lexical competition reached significance as a predictor (*t* = 2.603, *p* = 0.005) compared to lexical frequency (*t* = −0.094, *p* = 0.925). A significant regression equation was found when only lexical competition was included in the regression (*F*_(1,70)_ = 7.033, *p* = 0.005, *R*^2^ = 0.091). The PrAN decreased 0.302 standard deviations for each unit increase of lexical competition. The equation for the predicted amplitude is shown in example 2.

(2) PrAN = 0.302 (lexical competition)

### Regression Experiment 3

**Figure 4:**
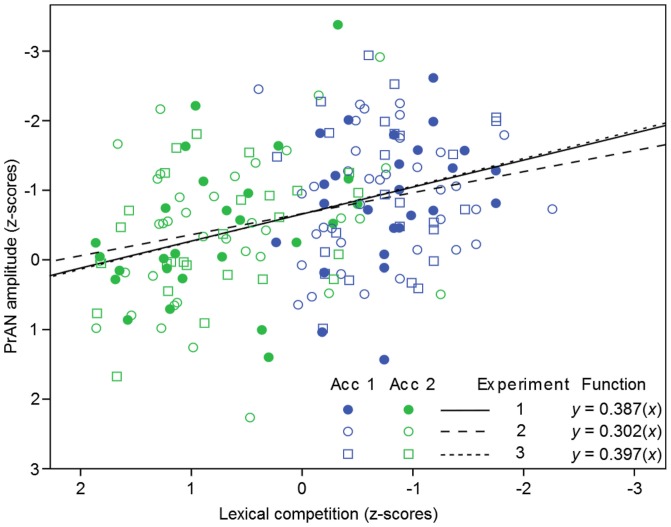
**Linear regression lines for the three experiments.** The solid line and dots represent data from Experiment 1, the dashed line and round unfilled dots represent data from Experiment 2, and the dotted line and squares represent data from Experiment 3. The plot shows that PrAN amplitudes became more negative with decreasing lexical competition in all experiments, corroborating the hypothesis in the present study.

Left anterior and left central electrodes were used for the regression analysis of Experiment 3 in the 220–280 ms time window, where Roll (2015) found a PrAN for word accents. The initial regression analysis involving both lexical frequency and competition was significant (*F*_(2,101)_ = 6.468, *p* < 0.002, *R*^2^ = 0.114). Again, lexical frequency was not significant (*t* = 1.024, *p* = 0.308). However, the effect of lexical competition was significant (*t* = 1.694, *p* = 0.047). In order to test the effect of lexical competition alone, this measure was included in a simple regression analysis. A significant regression equation was found (*F*_(1,50)_ = 9.370, *p* = 0.002, *R*^2^ = 0.158). The ERP negativity decreased 0.397 standard deviations for each unit increase of lexical competition. The equation for the predicted PrAN amplitude is shown in example 3. The standardized data from the three studies, as well as their regression coefficients, are presented in Figure 4.

(3) PrAN = 0.397 (lexical competition)

## Discussion

Results from the analysis of data from three experiments indicate that WIFs with few lexical competitors give rise to a PrAN in the ERPs between 136 and 280 ms. This is in line with the hypothesis that the negativity reflects the strength of pre-activation of upcoming material: WIFs which cue a smaller number of possible continuations (suffixes, simplex words or compounds) elicit a larger PrAN. Results show that the predictive certainty of how a word will end will be greater if the set of competing word forms is relatively small. Furthermore, linear regression analyses found that the PrAN could be calculated as a function of the number of competitors: the smaller the number of words that were possible based on a particular WIF, the greater the PrAN was. Thus, the effect appears to be gradual, in that it increases with decreased lexical competition.

The present findings suggest an explanation for the early negativity previously found for accent 1 words. Accent 1 WIFs have here been found to have fewer lexical competitors than accent 2 fragments, with accent 2 WIFs being associated with almost 11 times as many lexical items as compared to accent 1. A considerable overlap in time-course and topographical distribution between the effects of word accents and lexical competition was also observed. Further, the present results show a more fine-grained difference where the degree of lexical competition for word fragments associated with both word accents correlates with the degree of negativity in the effect previously reported for accent 1 words compared to accent 2 words.

A possible interpretation of the PrAN is that it reflects the activation of memory traces of possible word continuations (see Pulvermüller and Shtyrov, 2003). It might, however, be argued that the PrAN is simply an effect of lexical competition involving the word stem. For example, one could argue that the effect should in fact be interpreted as a positivity which increases with increasing lexical competition. However, viewing the PrAN as instead reflecting upcoming word structure receives support from a number of findings in previous studies. Combined fMRI/EEG results (Roll et al., 2015) showed that the amplitude of the PrAN correlated with activity related to morphological processing, suggesting that the PrAN might reflect the processing of a strongly expected upcoming suffix before it has been heard (since the effect is present on the word stem and ends before the suffix is present in the input). Furthermore, accent 1 stems seem to be associated with increased neural activity as indicated by both gRMS and BOLD effects, which would be unexpected in light of previous research on lexical competition effects in the brain (see e.g., Righi et al., 2010). In addition, the PrAN has been found for pseudo-stems which carry no lexical meaning (Söderström et al., 2016) indicating that phonological information in the WIF, e.g., tones, can be sufficient to activate suffixes. Lexical frequency, however, seems not to have any significant effect on the amplitude of the PrAN. Consequently, it seems unlikely that the negativity would simply be an effect of lexical competition, but rather that it reflects a relationship between WIFs with predictively charged segmental and suprasegmental features and their anticipated word-internal continuations. In Söderström et al. (2016), correlations between ERP amplitude and behavioral measures (such as accuracy) were only found for accent 1 stems, again suggesting that the effect is a negativity rather than a positivity. However, it is unlikely that lexical competition as defined in the present study is the only factor influencing the amplitude of the PrAN. Further studies are needed to disentangle other factors which could have an effect on PrAN amplitude, such as e.g., word probability due to discourse context.

While the present study investigated Swedish—in which associations between word onsets and possible continuations can be calculated in a fairly straightforward manner—it is highly probable that a similar mechanism will be found in other languages (see Dikker and Pylkkänen, 2013), given that they can be expected to have regularities and contexts (such as WIFs) which are constraining enough to narrow down the predictive space so as to enable pre-activation.

### Conclusion

We have described a negatively charged brain potential whose amplitude is inversely proportional to the number of possible word-internal continuations that can follow a WIF. It is suggested that this PrAN can be viewed as an index of the predictive certainty as to how a word is going to end. While much research on predictive processes in language processing has focused on the effects of e.g., prediction error, arising after the critical item has been heard or seen (e.g., N400/P600), we propose that PrAN rather reflects the pre-activation of lexical information (such as grammatical affixes or other possible continuations) *before* it is encountered.

The method used here, combining EEG data from WIF processing and lexical competition measures, has been shown to provide an insightful approach to investigating the very early integration of tonal and segmental cues in pre-activating word-internal lexical information in online speech comprehension.

## Ethics Statement

Participants gave their written informed consent to take part in the study, which was carried out in accordance with the Declaration of Helsinki. The research did not require review by an ethical board according to Swedish law, since it involved neither sensitive personal data, nor physical or psychological intervention, nor was it carried out on biological samples from physical persons.

## Author Contributions

PS was responsible for study planning and design, method development, data analysis and interpretation of results. MH contributed to the interpretation of the results, drafting and revising the manuscript. JF contributed to method development. MR contributed in study planning and design, method development and interpretation of results.

## Funding

The work was supported by the Swedish Research Council (Grant No. 2011–2284), Knut and Alice Wallenberg Foundation (Grant No. 2014.0139), Marcus and Amalia Wallenberg Foundation (Grant No. 2014.0039) and an infrastructure grant from the Swedish Research Council (SWE-CLARIN, 2014–2018; Grant No. 821-2013-2003).

## Conflict of Interest Statement

The authors declare that the research was conducted in the absence of any commercial or financial relationships that could be construed as a potential conflict of interest.

## Footnotes

^1^In the Roll et al. (2015) study, accent 1 stems elicited increased global root mean squares (gRMS) amplitude as compared to accent 2 stems, as well as an increased BOLD (blood-oxygen-level dependent) effect. As gRMS can be thought to reflect increased neural activity (Skrandies, 1990), we follow Roll et al. (2015) in analyzing the ERP effect as a negativity in response to accent 1 stems, rather than as a positivity for accent 2 stems.

^2^In South Swedish, the association between stem tones and compound words is not as straightforward as in Central Swedish, since some compounds are associated with accent 1. However, accent 2 is more common in compounds in South Swedish (Bruce, 1973; Frid, 2000).

^3^Please refer to the original studies for more details regarding EEG data acquisition and stimuli.

^4^http://spraakdata.gu.se/parole/lexikon/swedish.parole.lexikon.html
